# Further validation of the European and Brazilian Portuguese short version of the Postpartum Depression Screening Scale-7

**DOI:** 10.1192/j.eurpsy.2022.812

**Published:** 2022-09-01

**Authors:** A.T. Pereira, M. Barros, M. Aguiar, J. Azevedo, M.J. Soares, F. Carvalho, D. Pereira, A. Macedo

**Affiliations:** 1 Faculty of Medicine of University of Coimbra, Institute Of Psychological Medicine, Coimbra, Portugal; 2 State University of Southwest Bahia, Department Of Natural Sciences, Bahia, Brazil; 3 Federal University of Bahia, Postgraduate Program In Health Psychology, Bahia, Brazil

**Keywords:** perinatal mental health, PDSS, Postpartum

## Abstract

**Introduction:**

We have recently validated the Portuguese shortest version of the Perinatal Depression Screening Scale-PDSS-7 (items selected from the PDSS-21; each one representing a dimension evaluated by the PDSS-35), for the assessment of depression severity in pregnancy, both in Portugal and Brazil.

**Objectives:**

To analyze the validity and reliability of the PDSS-7 Portuguese version to evaluate postpartum women both from Portugal and Brazil.

**Methods:**

The Portuguese sample was composed of 304 women between the 2nd-6th postpartum months (Mean=20.09±7.21 weeks postpartum). These participants were not the same who participated in the psychometric study that led to the selection of the seven items. The Brazilian sample was composed of 121 women (Mean=10.51±4.53 weeks postpartum). All the participants completed the European/Brazilian Portuguese versions of PDSS-21, which was composed of the same items and included the seven items of PDSS-7. Participants also filled in the validated versions of Perinatal Anxiety Screening Scale and Profile of Mood States.

**Results:**

Confirmatory Factor Analysis revealed that the unidimensional model of PDSS-7 presented acceptable/good fit indexes in both samples (Portuguese/Brazilian: χ^2^/d.f.=2.6598/1.7897; RMSEA=.0740/.0807, CFI=.8289/.7934, TLI=.7901/.8434, GFI=.9298/.9496; p<.001). The PDSS-7 Cronbach’s alphas were of .841/.856 and all the items contributed to the internal consistency. Pearson correlations with postpartum anxiety (.646/.763) and negative affect (.666/.676) were significantly (p<.01) high. PDSS-7 mean scores were higher in the Brazilian sample (16.06±7.39 versus 11.37±4.37, p<.01).

**Conclusions:**

PDSS-7 presented validity (construct and convergent), reliability and utility in clinical and research settings, including in transcultural studies, in Portugal and Brazil, namely in the postpartum.

**Disclosure:**

No significant relationships.

